# Treatment for Osteoporosis among Women in Japan: Associations with Patient Characteristics and Patient-Reported Outcomes in the 2008–2011 Japan National Health and Wellness Surveys

**DOI:** 10.1155/2014/909153

**Published:** 2014-12-23

**Authors:** Masayo Sato, Jeffrey Vietri, Jennifer A. Flynn, Saeko Fujiwara

**Affiliations:** ^1^Eli Lilly K.K., Lilly Research Laboratories, Kobe 651-0086, Japan; ^2^Kantar Health, Health Outcomes Practice, Via Paleocapa 7, 20121 Milan, Italy; ^3^Hiroshima Atomic Bomb Casualty Council, Hiroshima 730-0052, Japan

## Abstract

This study was conducted to identify characteristics associated with treatment for osteoporosis among women aged 50 years and older in Japan and to explore differences among patients according to treatment regimen. Data were provided by a large annual survey representative of Japanese aged 18 and older; all measures were by self-report. Women aged 50 and older who reported diagnosed osteoporosis (*N* = 900) were compared based on current treatment status using bivariate statistics and logistic regression. Approximately 1 in 3 women in this study reporting diagnosed osteoporosis were currently untreated. Factors associated with current treatment for osteoporosis included having ≥1 physician visit in the prior 6 months (OR = 5.4, *P* < 0.001), self-rated moderate or severe osteoporosis (OR = 2.8, *P* < 0.001), completion of menopause (OR = 1.6, *P* < 0.05), and family history of osteoporosis (OR = 1.5, *P* < 0.05), while longer duration of osteoporosis diagnosis (OR = 0.9, *P* < 0.05) and arthritis (OR = 0.7, *P* < 0.05) were associated with lower odds of treatment. These findings suggest that diagnosed patients are not being actively managed in the longer term, and efforts need to be made to ensure that patients stay engaged with their healthcare providers.

## 1. Introduction

Osteoporosis is characterized by low bone mass density (BMD) and increased likelihood of bone fracture [1]. Fracture sequelae include pain, stature changes, decreased independence, psychiatric distress, increased hospitalization, and increased morbidity and mortality [2, 3]. Population-based estimates indicate that greater than 15 million people are affected by osteoporosis in Japan, with billions ($US) spent in Japan for hospital care of fractures [4].

Epidemiological estimates of the annual incidence of osteoporosis in Japan are 0.6% for men and 2.3% for women aged 40–79 [1]. Fracture rates indicate the burden of the disease on society, with hip fracture incidence increasing 1.7-fold from 1987 to 1997. With incidence of osteoporotic hip fractures in Japan estimated at approximately 31,300 men and 116,800 women per year [5], the 10-year probability of hip fracture is greater in Japan than in China or Korea [6–8].

The reduction of BMD with age and after menopause makes women older than 50 years a particularly vulnerable group. Residual lifetime risk of hip fracture in those 50 years of age in the Tottori prefecture, Japan, was recently estimated at 5.6% for men but 20.0% for women [6]. Vertebral fracture risk is also high in Japanese women, at 59.7 per 1,000 person-years for women aged 60–69 and 141 per 1,000 person-years in women aged more than 80 years [9]. Incidence of multiple vertebral fractures (i.e., vertebral fracture cascades) is higher in 65–75-year-old Japanese women than same-age nonnative Japanese women or Caucasian women [10, 11]. Moreover, vertebral fracture cascades confer particularly poor health outcomes (e.g., chronic pain, kyphosis, difficulties in performing daily activities, and death) relative to single vertebral fractures or other fractures [12]. The problem is likely to worsen in the future, as an increase is expected in the number of people older than 50 years old as the population ages, as well as a continued shift of population from rural areas where exposure to sunlight facilitates production of endogenous vitamin D to urban areas with less sun exposure [4].

However, pharmacotherapy is efficacious in slowing or halting bone loss, maintaining skeletal health, and reducing fracture risk. Common therapies include bisphosphonates and selective estrogen receptor moderators (SERMs), such as raloxifene, and calcitonin. These antiresorptive drugs reduce bone turnover by inhibiting osteoclastic activity, thus improving BMD and bone microarchitecture [13]. Active vitamin D_3_ is also frequently prescribed. A recent population-based study in Japan estimated the prevalence of vitamin D insufficiency at 81.3% and deficiency at 1.2% [14]. In the elderly, low vitamin D increases risk for falls which precipitate fracture. Due to these high insufficiency/deficiency rates, treatment guidelines indicate active vitamin D_3_ pharmacotherapies, which are more commonly used in Japan than elsewhere [1, 13].

Algorithms can be used to help determine osteoporotic fracture risk and therefore whether to initiate pharmacotherapy (e.g., Fracture Risk Assessment Tool, FRAX). Variables generally considered in such algorithms include age, gender, BMD, experience of previous fracture, site of previous fracture, family history of osteoporosis, body mass index (BMI), use of glucocorticoid medication, alcohol use, and tobacco use [1, 15, 16]. The relationship between experience of a previous fracture and risk for future fractures is strong but the follow-up and treatment of those who have experienced osteoporotic fractures are often considered inadequate. Improving secondary-fracture prevention is thus a current focus of a campaign by the International Osteoporosis Foundation (“Capture the Fracture” [4, 17]).

Despite availability of efficacious pharmacotherapy, a low proportion of those suffering osteoporotic fractures in Japan—as elsewhere—are actually treated prior to fracture, and some who experience an osteoporotic fracture do not receive follow-up treatment for osteoporosis [15, 18, 19]. Identifying relationships between patient characteristics and osteoporosis treatment status may help to elucidate which patients are more or less likely to receive treatment, and whether factors generally considered when evaluating fracture risk are also associated with receiving treatment. Awareness of factors associated with undertreatment may allow for better targeting of efforts to increase treatment uptake. Another gap in our knowledge regards data on patient-reported health outcomes according to type of treatment. Little is known, for example, on whether there is a relationship between the type of osteoporosis pharmacotherapy received and health outcomes in real-world patients in Japan.

With this background, the primary study objective was to identify health-related and sociodemographic personal characteristics associated with current osteoporosis treatment among Japanese women (50 years and older) diagnosed with osteoporosis. A secondary, exploratory objective was to describe patient-reported outcomes according to type of pharmacotherapy to generate hypotheses for future research.

## 2. Methods

The current study used data from the 2008 (*N* = 20, 000), 2009 (*N* = 20, 573), 2010 (*n* = 25, 000), and 2011 (*N* = 30, 000) Japan National Health and Wellness Surveys (NHWS; Kantar Health, New York, NY), an annual, cross-sectional study of individuals aged 18 years or older in Japan. The NHWS includes information related to diagnosis and treatment of a broad variety of conditions, health-related attitudes, health risk behaviors, and health-related outcome measures. Potential respondents to the NHWS are recruited through an existing web-based consumer panel, which recruits its members through opt-in emails, coregistration with panel partners, e-newsletter campaigns, banner placements, and both internal and external affiliate networks. All panelists explicitly agreed to be a panel member, registered with the panel through a unique email address, and completed an in-depth demographic registration profile.

The sample for NHWS is selected from this panel using a stratified random sample framework with quotas based on gender and age. Previous research has found the demographic composition of the Japan NHWS to be comparable to that of the Japanese adult population on important parameters [20]. Because sampling for NHWS is without regard to previous participation, a given respondent may have participated in more than one survey during the four-year period reviewed here. Only the most recent data for such individuals were included so as to avoid nonunique responses. All respondents to NHWS provided informed consent, and each of the annual surveys was approved by Essex Institutional Review Board (Lebanon, NJ). Because of the focus on treatment within diagnosed osteoporosis, only women aged 50 and older who reported a physician diagnosis of osteoporosis were included in the present study.

All information was collected through self-report. Variables collected in the NHWS which were of interest for the comparison between treated and untreated patients included sociodemographic and health characteristics, patient characteristics specific to osteoporosis, fracture risk factors, and healthcare resource use. Sociodemographic characteristics included age, household income, marital status, and level of education. General health characteristics included body mass index, use of alcohol, cigarettes, exercise, use of oral glucocorticoid medications, and comorbidity burden according to the Charlson comorbidity index (CCI) [21]. Factors specific to osteoporosis included self-rated severity of osteoporosis, length of diagnosis, and whether the respondent had received a BMD scan. Known risk factors for fracture not already mentioned above included previous fracture since age 50, family history of osteoporosis, back pain, and arthritis. Healthcare resource use variables included whether the individual visited a physician in the prior six months, whether she made a visit to the emergency room in the prior 6 months, and whether she was admitted to the hospital in that time.

The exploratory comparison of outcomes included scores from the revised Medical Outcomes Study 12-Item Short Form Survey Instrument (SF-12v2), a multipurpose, generic instrument comprised of 12 questions [22]. This instrument can be used to summarize functional health by two summary scores, the physical component summary (PCS) and mental component summary (MCS). Each score has a mean of 50 and a standard deviation of 10 for the Japanese population [23], with higher scores indicating better health. Several of the items from the SF-12v2 can be used to generate a health state utility score, the SF-6D. The SF-6D is a preference-based single index measure for health using general population values [24]. The SF-6D index has interval scoring properties and yields summary scores on a theoretical 0-1 scale (with an empirical floor of 0.3). Higher scores indicate better quality of life. Ratings of impairment in nonwork activities (activity impairment) from the Work Productivity and Activity Impairment questionnaire were also included. This measure yields a percentage from 0 to 100% with higher ratings indicating more health-related impairment [25]. The numbers of physician visits, emergency room (ER) visits, and hospitalizations in the prior 6 months were also collected from the survey.

### 2.1. Analysis

The primary objective of identifying patient characteristics associated with current treatment was addressed by comparing women currently being treated for osteoporosis with those not currently treated, using chi-square for categorical variables and *t*-test for continuous variables. This was followed by binary logistic regression to assess which patient characteristics were associated with higher adjusted odds of current use of prescription treatment for osteoporosis when considered simultaneously.

To explore differences in outcomes according to type of treatment, the group currently receiving treatment for osteoporosis was further subdivided according to the primary type of medication used. Four groups were constructed to allow for inclusion of common treatment patterns while excluding more complicated combinations of medications that would have smaller sample sizes and reflected the treatment patterns observed in the sample rather than official recommendations (i.e., [1]). The groups were raloxifene (the only SERM used by respondents in this sample) without a bisphosphonate, bisphosphonate (alendronate, minodronic acid, risedronate, or zoledronic acid) without raloxifene, active vitamin D_3_ (alfacalcidol, calcitriol) without other osteoporosis medication, and calcitonin without other osteoporosis medications. Respondents in the raloxifene and bisphosphonate categories were still included in these categories if using active vitamin D_3_, as prescribing these medications in combination with another osteoporosis medication is common practice in Japan. Patient characteristics and outcomes were compared using one-way ANOVA for continuous variables and chi-square for categorical variables. The outcomes of the treatment groups were also compared using generalized linear models incorporating treatment group along with age, household income, BMI category, length of diagnosis, and CCI. Models of health-related quality of life incorporated a normal probability distribution and identity function. Models of activity impairment and healthcare use specified a negative binomial distribution and a log-link function. A 5% (two-tailed) alpha error rate was adopted for all null-hypothesis tests; no adjustments were made for multiplicity.

## 3. Results

Response rates for the NHWS Japan surveys providing data for this study were 40.0%, 22.7%, 24.9%, and 15% in 2008 through 2011, respectively. A total of 17,722 unique women aged 50 and older were identified, and 900 (5%) reported physician diagnosis of osteoporosis. These respondents with osteoporosis were approximately 67 years old on average (range 50 to 92) and had been diagnosed for a mean of 5.3 years. The majority (65.1%) of respondents diagnosed with osteoporosis were currently being treated with a prescription medication. Most reported completing menopause (75.4%) and having previously had a BMD scan (89.4%). Fewer than half reported a fracture since age 50 (37.3%), and a similar number reported their osteoporosis was moderate or severe (41.2%) as opposed to mild.

Unadjusted comparisons showed a variety of differences in the characteristics of patients by treatment status (Table 1). Those who were currently treated were slightly older on average, more likely to have completed menopause, more likely to report moderate or severe osteoporosis, and more likely to have visited a physician in the prior 6 months. There were also trends that approached significance (*P* < 0.10), with those currently treated for osteoporosis potentially having a greater likelihood of reporting daily alcohol use, reporting vigorous exercise in the previous month, to currently use glucocorticoid medication, to have a family history of osteoporosis, and to report a fracture since age 50 relative to those diagnosed but not currently treated.

Twenty-three respondents were excluded from the regression due to missing data for length of diagnosis. The multivariable logistic regression revealed that several personal and disease characteristics were associated with greater odds of current treatment (Table 2), though the pattern of significant variables differed somewhat from the bivariate analysis. Factors significantly associated with current treatment included having completed menopause, having a family history of osteoporosis, reporting moderate or severe osteoporosis (relative to mild), and having visited a physician in the prior 6 months. Lower adjusted odds of treatment were associated with arthritis and longer duration of diagnosis. There was also a trend for those who were not sure of having a BMD scan to be less likely to be treated relative to those who did have a scan (*P* = 0.052). No other variables approached significance in this regression (all *P* > 0.10).

The category definitions were chosen for the exploratory comparison of treatments provided for inclusion of 77% (450/586) of respondents currently using osteoporosis medications in the sample and 50% of the total sample reporting a physician diagnosis of osteoporosis. Patient characteristics according to type of treatment are presented in Table 3. There were no significant differences between the groups, though there was a trend (*P* = 0.065) for cigarette smoking to differ across treatment groups, with raloxifene users having a very high proportion of respondents who indicated that they never have smoked cigarettes.

Bivariate comparison of unadjusted outcomes by type of treatment is presented in Table 4. Only differences in mean MCS scores across the groups approached statistical significance (*P* < 0.10), with no differences reaching the critical level of *P* < 0.05. Generalized linear models of health outcomes excluded 8 respondents due to missing data for duration of diagnosis. After adjustment for age, household income, BMI category, length of diagnosis, and CCI, there was a significant relationship between treatment group and MCS scores (*P* < 0.05). Treatment group was not significant in the models of other outcomes (Table 5).

## 4. Discussion

Study findings showed that approximately one-third of women already diagnosed with osteoporosis in Japan aged 50 and older contacted during four years of data collection were not currently using a prescription. This suggests that many women who would likely benefit from osteoporosis treatment are not receiving it. The factor most strongly related to current treatment among diagnosed women was report of a physician visit in the prior 6 months, suggesting that regular follow-up with healthcare providers may be a key factor in determining whether or not women receive osteoporosis treatment. This finding is consistent with the result for duration of diagnosis; after taking into account other relevant variables, longer duration of diagnosis was associated with lower odds of treatment. This is consistent with previous research on persistence and adherence to osteoporosis medications, which are low both inside and outside Japan [26–30]. Other significant correlates of treatment status corresponded with identified risk factors for fracture, including menopausal status, family history of osteoporosis, and perceptions of more-severe osteoporosis. It is a positive sign that women with these risk factors for fracture are being treated.

However, other fracture risk variables were not associated with treatment status, most notably history of previous fracture. As reported above, the association of previous fracture with an increased risk of future fracture has been widely documented, and secondary fracture prevention is the focus of the IOF's “Capture the Fracture” campaign [4, 17]. Thus, these results suggest that identifying and treating those with previous fracture may need to be a higher priority for physicians in Japan than it has been in the past. Similarly, age has a strong and clear association with fracture risk [15] but was unrelated to treatment status in this study. However, this may be an expected finding, as in Japan the intervention thresholds take age into account, allowing for higher risk among older patients before the intervention threshold is reached [15]. Other risk factors which were not significantly related to treatment in the present study were current smoking, alcohol use, and use of oral glucocorticoids. However, while included in pharmacotherapy treatment decision tools, the associations between smoking and alcohol use with fracture risk are of a much smaller magnitude than previous fracture [15]. As very few people were on glucocorticoids, the null finding may be the result of insufficient power. Interestingly, those who reported comorbid arthritis were less likely to be currently treated for osteoporosis. One potential explanation is that such patients may have had higher BMD than respondents without arthritis, thereby affecting patients' and/or physicians' perceptions of the urgency of treatment. Osteoarthritis has been associated with higher BMD, though the relationship between osteoarthritis and osteoporosis is still not well understood [31, 32]. We can only speculate on this point as BMD results were not available for survey respondents.

Outcomes were not statistically different across treatment groups excepting MCS scores in the adjusted comparisons. Nevertheless, if replicated in a larger sample, the numeric differences in the mean values of some outcomes would be considered important (i.e., equal to or greater than the minimally important difference [33]). Likewise, the pattern of means also suggests a relationship between treatment type and physician visits that may be worthwhile to explore in further research. It is also important to bear in mind that the significance level was not adjusted to account for multiple comparisons. Indeed, if the Bonferroni correction were applied to maintain 5% experiment-wise alpha error across the multivariable comparison of the seven outcomes considered here, the critical value would be 0.0071, lower than any of the observed *P* values for treatment type.

The present study should be considered in light of the limitations of the methodology. Clinical information about previous treatment or clinical measures such a BMD scan results was not available and would likely explain additional variance in treatment status. The analysis was correlational and cross-sectional and, as is the case for all correlational analyses, directionality of the associations could not be determined. Some variables, such as perceived severity, could have been effects rather than causes of treatment. We were unable to examine composite fracture risk for individual patients, which would have potentially been more valuable than individual risk factors. Survey questions did not allow us to distinguish between undertreatment, problems with treatment initiation, and lack of persistence, which have all been shown to be significant problems in osteoporosis treatment in other populations [26–30].

Response rates to the survey were modest to low, which may have introduced some self-selection bias. It is possible that individuals more invested in, or highly conscious of, their health would be more likely to respond to the study survey. It is not fully clear how such a bias might affect the results. However, the most likely effect would be overestimation of the proportion of women currently treated relative to the actual situation in Japan, as this is a point estimate which would be sensitive to potential differences between the responders and nonresponders. It is less clear how the low response rate may have affected the relationships between patient characteristics and treatment status or the exploratory analysis comparing types of treatments. These comparisons were within the same sample (i.e., all were responders to the survey), and so comparison groups would share the same bias rather than having the bias confounded with analysis groups.

As with any self-report survey, measurement error could have been introduced by recall biases or errors. The sample sizes for the exploratory comparison of outcomes by type of treatment were limited, which may have prevented the detection of differences in the treatment groups that could be detected in a larger sample. Finally, the limitation of cross-sectional analysis is also applicable to the comparisons of outcomes, and the difference in MCS scores may be a consequence of treatment choice, a cause of treatment choice, or a spurious finding. As previously noted, the exploratory nature of the analysis included numerous hypothesis tests without adjustment for type 1 error.

In summary, approximately one in three women included in this study was not currently being treated with a prescription medication for osteoporosis despite an average length of diagnosis of nearly 6 years. Treatment status was associated with some, but not all, established fracture risk factors. The patient characteristics most strongly associated with current treatment according to the adjusted odds ratios were a recent physician visit and a perception of more-severe osteoporosis, both of which suggest that concern about the disease and contact with a healthcare provider facilitate treatment, while objective risk factors such as age and fracture history were not strongly associated with current treatment. These findings suggest that diagnosed patients are not being actively managed in the longer term, and efforts need to be made to ensure that patients stay engaged with their healthcare providers.

## Figures and Tables

**Table 1:** Characteristics of women in Japan diagnosed with osteoporosis.

	Osteoporosis treatment status						
	Total (*N* = 900)		Currently treated (*N* = 586)		Not currently treated (*N* = 314)		
	Mean	SD	Mean	SD	Mean	SD	*P* value^1^

Age (years)	66.9	7.45	67.3	6.9	66.2	8.3	0.033
CCI	0.30	1.54	0.34	1.87	0.23	0.55	0.285
Length of diagnosis (years)	5.3	5.5	5.1	5.5	5.7	5.6	0.127

	*n*	%	*n*	%	*n*	%	*P* value^2^

Married/living with partner	627	69.7%	409	69.8%	218	69.4%	0.909
University degree	150	16.7%	97	16.6%	53	16.9%	0.900
Household income							0.848
Below median	472	52.4%	305	52.0%	167	53.2%	
Above median	341	37.9%	222	37.9%	119	37.9%	
Decline to answer income	87	9.7%	59	10.1%	28	8.9%	
Cigarette smoking							0.317
Never	694	77.1%	449	76.6%	245	78.0%	
Current	87	9.7%	53	9.0%	34	10.8%	
Former	119	13.2%	84	14.3%	35	11.1%	
Daily alcohol use	68	7.6%	51	8.7%	17	5.4%	0.075
Exercise	504	56.0%	342	58.4%	162	51.6%	0.051
BMI categories							0.212
Underweight	115	12.8%	76	13.0%	39	12.4%	
Normal	676	75.1%	446	76.1%	230	73.2%	
Overweight or obese	90	10.0%	50	8.6%	40	12.7%	
Declined to answer weight	19	2.1%	14	2.4%	5	1.6%	
Completed menopause	679	75.4%	463	79.0%	216	68.8%	0.001
On glucocorticoids	41	4.6%	32	5.5%	9	2.9%	0.075
Back pain	113	12.6%	78	13.3%	35	11.1%	0.350
Arthritis	160	17.8%	98	16.7%	62	19.7%	0.258
Family history of osteoporosis	194	21.6%	137	23.4%	57	18.2%	0.069
BMD scan							0.026
Yes	805	89.4%	536	91.5%	269	85.7%	
No	58	6.4%	31	5.3%	27	8.6%	
Do not know	37	4.1%	19	3.2%	18	5.7%	
Previous fracture since age 50	336	37.3%	232	39.6%	104	33.1%	0.056
Moderate or severe osteoporosis	371	41.2%	287	49.0%	84	26.8%	<0.001
Visited physician	829	92.1%	565	96.4%	264	84.1%	<0.001
Visited ER	68	7.6%	43	7.3%	25	8.0%	0.736
Hospitalized	82	9.1%	57	9.7%	25	8.0%	0.380

^1^Independent-samples *t*-test; ^2^Pearson chi-square test; CCI: Charlson comorbidity index; BMD: bone mineral density; BMI: body mass index; ER: emergency room.

**Table 2:** Adjusted odds ratios of current treatment among women aged 50 and older in Japan with diagnosed osteoporosis (*N* = 877).

Factor	OR	95% confidence interval		*P* value
		Low	High	
Age (5-year increment)	1.045	0.924	1.181	0.486
Married/living with partner	1.080	0.763	1.529	0.665
University degree	1.015	0.665	1.550	0.945
Household income				
Low income	Reference			
High income	1.176	0.835	1.657	0.353
Declined to answer income	1.265	0.733	2.185	0.399
Cigarette smoking				
Never smoker	Reference			
Current smoker	0.810	0.476	1.379	0.438
Former smoker	1.321	0.827	2.110	0.244
Daily alcohol use	1.654	0.868	3.152	0.126
Exercise	1.048	0.761	1.443	0.774
BMI category				
Underweight	1.162	0.727	1.859	0.530
Normal weight	Reference			
Overweight or obese	0.719	0.435	1.19	0.199
Declined to answer weight	1.852	0.585	5.864	0.295
CCI	1.028	0.87	1.214	0.747
Completed menopause	1.587	1.084	2.323	0.018
On oral glucocorticoids	2.106	0.841	5.269	0.112
Back pain	1.237	0.761	2.011	0.390
Arthritis	0.654	0.435	0.982	0.040
Family history of osteoporosis	1.472	1.001	2.164	0.049
BMD scan history				
Has had BMD scan	Reference			
Never had BMD scan	0.749	0.405	1.385	0.357
Not sure of BMD scan	0.473	0.222	1.007	0.052
Previous fracture since age 50	1.26	0.905	1.756	0.172
Moderate or severe osteoporosis (relative to mild)	2.777	1.991	3.873	<0.001
Duration of osteoporosis (5-year increment)	0.857	0.745	0.986	0.031
Healthcare use				
Visited physician (past 6 months)	5.374	3.007	9.604	<0.001
Visited ER (past 6 months)	0.842	0.456	1.555	0.583
Hospitalized (past 6 months)	0.926	0.513	1.670	0.798

CCI: Charlson comorbidity index; BMD: bone mineral density; ER: emergency room.

**Table 3:** Patient characteristics according to type of osteoporosis treatment (*N* = 450).

	Raloxifene		Bisphosphonates		Active vitamin D_3_ alone		Calcitonin alone		
	(*N* = 46)		(*N* = 298)		(*N* = 60)		(*N* = 46)		
	Mean	SD	Mean	SD	Mean	SD	Mean	SD	*P* value^1^

Age	66.5	7.0	67.1	6.9	68.4	7.5	66.8	8.0	0.490
CCI	0.15	0.36	0.41	2.53	0.47	1.02	0.20	0.50	0.787
Length of diagnosis	4.6	4.4	5.2	5.8	5.6	6.0	4.9	5.1	0.934

	*n*	%	*n*	%	*n*	%	*n*	%	*P* value^2^

Married/living with partner	37	80.4%	214	71.8%	39	65.0%	33	71.7%	0.382
University degree or greater	9	19.6%	48	16.1%	9	15.0%	6	13.0%	0.854
Household income									0.266
Below median	22	47.8%	155	52.0%	30	50.0%	27	58.7%	
Above median	14	30.4%	115	38.6%	24	40.0%	14	30.4%	
Declined answering	10	21.7%	28	9.4%	6	10.0%	5	10.9%	
Cigarette smoking									0.065
Never smoker	42	91.3%	227	76.2%	46	76.7%	35	76.1%	
Current smoker	2	4.3%	34	11.4%	3	5.0%	2	4.3%	
Former smoker	2	4.3%	37	12.4%	11	18.3%	9	19.6%	
Daily alcohol use	2	4.3%	34	11.4%	3	5.0%	2	4.3%	0.130
Exercise	31	67.4%	172	57.7%	32	53.3%	21	45.7%	0.185
BMI categories									0.994
Underweight	6	13.0%	38	12.8%	7	11.7%	6	13.0%	
Normal	34	73.9%	230	77.2%	47	78.3%	36	78.3%	
Overweight or obese	5	10.9%	23	7.7%	5	8.3%	4	8.7%	
Declined to answer	1	2.2%	7	2.3%	1	1.7%	0	0.0%	
Completed menopause	37	80.4%	233	78.2%	45	75.0%	36	78.3%	0.923
On glucocorticoids	1	2.2%	19	6.4%	3	5.0%	2	4.3%	0.671
Back pain	6	13.0%	38	12.8%	9	15.0%	5	10.9%	0.938
Arthritis	6	13.0%	56	18.8%	10	16.7%	10	21.7%	0.712
Family history of osteoporosis	11	23.9%	69	23.2%	17	28.3%	13	28.3%	0.770
Have you ever had a bone mass density test/scan?									0.720
Yes	43	93.5%	273	91.6%	55	91.7%	42	91.3%	
No	1	2.2%	15	5.0%	3	5.0%	4	8.7%	
Not sure	2	4.3%	10	3.4%	2	3.3%	0	0.0%	
Fracture since age 50	12	26.1%	131	44.0%	23	38.3%	20	43.5%	0.135
Moderate or severe osteoporosis	23	50.0%	138	46.3%	27	45.0%	24	52.2%	0.847
Visited physician (prior 6 months)	45	97.8%	286	96.0%	56	93.3%	45	97.8%	0.591
Visited ER (prior 6 months)	3	6.5%	24	8.1%	7	11.7%	4	8.7%	0.780
Visited hospital (prior 6 months)	4	8.7%	28	9.4%	6	10.0%	3	6.5%	0.926

^1^One-way ANOVA; ^2^Pearson chi-square test; CCI: Charlson comorbidity index; BMD: bone mineral density; BMI: body mass index; ER: emergency room.

**Table 4:** Unadjusted mean outcomes according to type of osteoporosis treatment.

	Raloxifene		Bisphosphonates		Active vitamin D_3_ alone		Calcitonin alone		*P* value^1^
	(*N* = 46)		(*N* = 298)		(*N* = 60)		(*N* = 46)		
	Mean	SD	Mean	SD	Mean	SD	Mean	SD	
HRQoL (SF-12v2)									
MCS	50.97	8.46	47.55	10.59	46.64	10.77	50.07	8.93	0.063
PCS	44.52	9.35	44.73	10.71	42.93	13.37	45.05	8.42	0.676
Health utility score (SF-6D)	0.745	0.139	0.718	0.141	0.703	0.151	0.756	0.135	0.163
Activity impairment (%)	28.91	27.26	32.38	29.15	34.50	29.08	31.09	28.85	0.788
6-month healthcare use									
Physician visits	10.59	8.48	15.41	16.76	17.20	19.16	12.57	14.47	0.130
ER visits	0.09	0.35	0.32	2.06	0.37	1.63	0.15	0.51	0.796
Hospitalizations	2.09	11.20	1.91	9.32	2.10	8.74	3.39	18.02	0.855

^1^One-way ANOVA; HRQoL: health-related quality of life; MCS: mental component summary; PCS: physical component summary; ER: emergency room.

**Table 5:** Regression-adjusted mean outcomes according to type of osteoporosis treatment.

	Raloxifene		Bisphosphonates		Active vitamin D_3_ alone		Calcitonin alone		*P* value^1^
	(*N* = 46)		(*N* = 291)		(*N* = 59)		(*N* = 46)		
	Adjusted mean	SE	Adjusted mean	SE	Adjusted mean	SE	Adjusted mean	SE	
HRQoL									
MCS	50.5	1.6	46.8	0.9	45.3	1.5	49.2	1.6	0.027
PCS	42.1	1.6	42.4	0.9	40.8	1.5	42.6	1.7	0.747
SF-6D	0.725	0.022	0.697	0.013	0.677	0.020	0.732	0.022	0.138
Activity impairment (%)	30.7	4.4	33.7	2.8	34.7	4.9	32.6	4.8	0.911
6-month healthcare use									
Physician visits	11.9	1.8	16.5	1.4	18.3	2.5	14.6	2.2	0.102
ER visits	0.2	0.2	0.3	0.1	0.3	0.2	0.4	0.4	0.916
Hospitalizations	4.2	3.6	2.0	0.8	1.2	1.1	1.7	1.4	0.700

^1^Wald chi-square for treatment group; HRQoL: health-related quality of life; MCS: mental component summary; PCS: physical component summary; ER: emergency room.
